# Experiences of vancomycin-resistant enterococci carriage – varying and insufficient information from healthcare providers and divergent feelings in everyday life: a qualitative study

**DOI:** 10.1016/j.infpip.2026.100551

**Published:** 2026-05-12

**Authors:** Karin Uggla, Robin Razmi, Josef D. Järhult, Kati Knudsen, Maria Lindberg

**Affiliations:** a Centre for Research and Development, Region Gävleborg/Uppsala University, Gävle, Sweden; b Department of Medical Sciences, Uppsala University, Uppsala, Sweden; c Department of Caring Sciences, Faculty of Health and Occupational Studies, University of Gävle, Gävle, Sweden

**Keywords:** Antimicrobial resistance, Infection prevention and control, Patient-professional interaction, Qualitative research, Vancomycin-resistant enterococci

## Abstract

**Background:**

Antibiotic-resistant bacteria pose a significant challenge in healthcare. Many studies have examined this concern from the perspective of healthcare personnel, highlighting hygiene precautions. Research on patients’ perspectives, particularly regarding vancomycin-resistant enterococci (VREs) carriership, remains limited. The aim of this study was to describe individuals’ experiences of living with VRE.

**Methods:**

In this study, a descriptive design was applied using qualitative content analysis. Twenty-two informants with VRE carriage participated in semi-structured individual interviews.

**Results:**

The main theme, *Life goes on despite the different circumstances* illustrates how VRE carriers navigate daily life. The informants reported that overall, VRE carriage had a minimal impact on their lives; however, it caused uncertainty, particularly in healthcare interactions.

**Conclusions:**

The findings showed that information about VRE carriage is unclear and contributed to uncertainty in healthcare contacts. Carriership, however, does not have a major impact on daily life. To ensure greater clarity, there is a need for increased knowledge of VRE and its management among healthcare personnel and an increased focus on person-centred care to improve healthcare interactions.

## Introduction

Antibiotic-resistant bacteria, such as vancomycin-resistant enterococci (VREs), are increasing worldwide [1]. Due to their resistance to several classes of antibiotics and their ability to spread among individuals with risk factors, these bacteria pose a significant problem in healthcare settings and in elderly care [2]. In Sweden, VRE is regulated under the Communicable Diseases Act, which mandates that carriers follow specific infection control guidelines to prevent transmission. There is currently no declassification protocol for VRE carriage; consequently, individuals who tested positive once will remain designated as carriers indefinitely, regardless of subsequent negative tests or lack of symptoms [3].

Horizontal transmission is an important contributing factor for the spread of VREs within healthcare facilities [4]. Thus, healthcare personnel are required to follow hygiene precaution using personal protective equipment when caring for patients [1,5]. Although VRE carriers are managed in single rooms with enhanced cleaning protocols and may require contact tracing, basic hygiene practices during patient interactions remain the same as for other patients [5]. According to Watson *et al.* [6], patients perceive that healthcare personnel do not always follow hygiene precautions and that healthcare personnel lack education about antibiotic-resistant bacteria. In a literature review, Morgan *et al.* [7] found four main adverse outcomes as unintended consequences of efforts to limit the spread of antibiotic-resistant bacteria. These included reduced patient–healthcare personnel contact, changes in systems of care that produce delays and more non-infectious adverse events, increased symptoms of depression and anxiety among patients, and decreased patient satisfaction with care.

In person-centred care [8,9], it is essential to provide patients with information and knowledge about their disease and to support their understanding, acknowledging that patients differ in their ability to absorb information [10,11]. Previous qualitative studies on experiences of carriership of antibiotic-resistant bacteria describe feelings of being worthless, dirty [12], overlooked by healthcare personnel [12,13], and inadequately informed about their status [14,15]. Moreover, a recent systematic review has shown that individuals with carriership faced depression and social isolation when hospitalized [16]. Existing literature has examined patients’ experiences of living with antibiotic-resistant bacteria and how these affect their daily lives [[12], [13], [14],[16], [17], [18]]. For example, in a study that included persons with carriership of extended-spectrum beta-lactamase (ESBL)–producing bacteria, Uggla *et al.* [17] described varying influences in everyday life, from anxiety, worry, and depression to no influence at all. Furthermore, the authors concluded a lack of information and uncertainty about carriership. However, studies directed towards perceptions of VRE carriership are sparse in the literature, and an increased knowledge about living with VRE carriage can enable improved patient treatment. Thus, the aim of this study was to describe individuals’ experiences of living with VRE in a Swedish context since concerns about antibiotic-resistant bacteria carriership have been reported in previous studies but not specifically among VRE carriers.

## Methods

The Standards for Reporting Qualitative Research [19] checklist was used as a guide to report this qualitative descriptive study [20].

### Setting and sample

This study was conducted in a county in mid-Sweden with approximately 288,000 habitants. All informants were recruited via the Infectious Disease Control Unit register using a purposive sampling procedure. Inclusion criteria were individuals aged >18 years diagnosed with VRE between 2010 and 2022. The only exclusion criterion was inability to communicate in Swedish. A research assistant contacted 36 eligible individuals by telephone. Of those, 30 agreed to be contacted by the first author (K.U.), who further informed and explained the aim and procedure of the study. A total of 22 individuals (15 men and 7 women) agreed to participate. Their mean age was 65 years (range: 49–75). Six were employed, 14 were retired, and two were disability retirees. The participants were diagnosed with VRE between 2013 and 2022 (n=5 within 2 years; n=2 within 2–6 years; n=15 >6 years ago).

### Data collection

Semi-structured individual interviews were conducted between February 2023 and January 2024. A written informed consent was obtained before the interview. An interview guide was used focussing on how VRE carriage has affected their daily life. Informants were encouraged to reflect on and exemplify situations that reminded them of their carriership. We used clarification prompts when necessary, e.g. asking ‘Could you elaborate?’ All interviews were performed by the first author (K.U.) in a place chosen by the informant. Nine of the interviews were conducted via telephone, eight by Skype, and five in-person. All interviews were audio recorded and transcribed verbatim into text files. The interviews lasted between 8 and 51 min (median: 17 min). The concept of information power guides sample size decisions in qualitative research, indicating that the more relevant information participants hold for the study aim, the fewer are needed [21]. Based on the research group’s experience in conducting qualitative research, combined with a clearly defined and narrow aim, purposive sampling from a well-specified target group, and the use of focussed dialogues, we estimated that 20 interviews would suffice. We were, however, given the opportunity to conduct 22 interviews, and the data were deemed rich and informative, aligning with the study aim.

### Data analysis

The data analysis was carried out by using Graneheim and Lundman’s method [22] in five steps:

1. The interview transcripts were read and re-read several times to find meaning units corresponding to the study aim.
2. In places where the informant described their experiences of living with VRE, the meaning units were marked and shortened to condensed meaning units.
3. The condensed meaning units were labelled with a code.
4. The codes were compared for differences and similarities and sorted into subcategories and subsequently into categories.
5. The transcribed interviews were re-read, and an abstracted theme was formulated describing how the informants talked about living with carriership.

Steps 1–3 were done by two of the authors (M.L. and K.U.), and steps 4 and 5 were thoroughly discussed in dialogue with all the co-authors until consensus was reached. No further aspects emerged in the analysis of the last interviews, and no further sampling was deemed necessary. We used tables in Microsoft Word (Microsoft 365 MSO, Version 2601) to sort the data during the analysis process. Three authors (M.L., K.U., and K.K.) had previous experience in interviewing and qualitative data analysis. All authors had previous experience of caring for patients with infectious diseases, which was actively considered during the analysis process to minimize interpretation bias. Verbatim quotes from the informants are presented to illustrate the findings.

## Findings

The theme *Life goes on regardless of the different circumstances* describes how individuals with VRE carriership experience their daily life. The informants reported minimal impact in everyday life as the carriage does not pose a major obstacle; however, it contributes to uncertainty, especially in healthcare contacts. The theme is supported by two categories and five subcategories as described in the following.

### Conflicting handling caused uncertainty about carriership

The category contains two sub-categories related to healthcare contacts. The informants described variations in the management they received during care situations, which reminded them of their carriership. Furthermore, they perceived unclear information about carriership.

#### Carriership becomes relevant during healthcare contacts

The informants’ descriptions of healthcare interactions varied. At clinic visits, some were not allowed in waiting rooms, while others were allowed. During hospital stays, some initially received private rooms, while others did not.

*‘I don’t get to sit in the regular waiting room and wait like the others; I receive special treatment. I’m given my own room. I never have to sit and wait – I’m placed immediately in a special room. That’s how it has been the times I’ve been to the emergency department in this case. When it comes to dental care, there wasn’t anything special other than that they were aware of it, so to speak’* (informant 3).

The informants perceived healthcare personnel as indifferent to carriership; some were fearful but relaxed once they learned the latest VRE test was negative. They also observed inconsistencies in the use of protective equipment, ranging from none to full gear (e.g. aprons, face masks, and gloves). Healthcare personnel sometimes wore protective equipment even when no physical examination was required. Informants explained that cleaning staff initially followed their usual routines and only adjusted them after feedback, creating uncertainty about their awareness of the VRE carriership.

Having a private room was described as an advantage, even though it served as a reminder of carriership. Informants noted infection risk notices on their doors, which was troublesome as people were afraid to go near the room. There were differences in the way healthcare personnel dealt with VREs, even in the same ward, which caused confusion. Moreover, the informants described uncertainty if VRE carriage was noted in their medical record, raising concerns about receiving proper treatment.

*‘Some act very strangely, and some don't care at all, so it simply depends on the staff … // … Yes, they are almost afraid that I will come near, but to me, it feels like they could get infected, and some don’t seem to take it seriously’* (informant 17).

#### Information from healthcare providers about carriage status varies

Informants expressed confusion about VRE carriage, particularly regarding its duration and implications, e.g. being initially told they carried VRE, only to later be informed otherwise, which increased uncertainty. Information from healthcare providers was often described as vague or insufficient, especially concerning the implications of VREs and recommended behaviour. Communication methods varied, including phone calls, hospital conversations with healthcare personnel, or letters containing only general information. However, some informants reported having no recollection of receiving any information.

*‘Because I had already submitted samples since I had amoeba … and then I submitted samples, but they said I didn’t have anything … I can’t even remember being informed that I have this [VRE]’* (informant 2).

Provided information was described as easy to forget after discharge, particularly because VRE carriage is asymptomatic. Informants believed information should be clearer and delivered through personal dialogue and suggested accessible resources such as health information websites. Many took it upon themselves to seek information via search engines regarding VRE severity, transmission, symptoms, and relapse risks. They also questioned persistent carriership despite negative test results, whether they could infect others while asymptomatic, and the safety of using hospital facilities. Overall, follow-up was described as inadequate, and informants requested repeated testing and opportunities to ask questions for clarification over time.

*‘It has been extremely inadequate; a letter was sent home … um … it came by post. It informed me that I had VRE and two information sheets about this … I haven’t met anyone. I’ve never received any more information about this at all … // … There was no opportunity to ask … um … specific follow-up questions, like what is this, how it affects you. So then you have to try to Google it, and I think that’s a bit bad’* (informant 3).

### No noticeable difference despite the situation

This category contains three sub-categories that relate to the informants’ actions and feelings in relation to carriership. Everyday life was mostly the same as before VRE diagnosis; however, carriership could result in emotional strain. Moreover, modified cleaning routines and hand hygiene measures were performed to limit infection spread to others.

#### Everyday life is mostly the same as before

The informants reported that carriership had not impacted their daily life or work situation significantly. They continued their usual routines, engaged in activities as before, and made no adjustments at home. One informant, for instance, still swam regularly without concern.

*‘I don’t really think about it at all … it’s only when I go to the hospital, if I’m admitted or something like that … that’s when I’m reminded of it more, so to speak. But otherwise, in everyday life and things like that, it doesn’t affect me … I don’t think about it. In that sense, I try to live as usual’* (informant 11).

Some were unaware of their VRE status, so it had not influenced their everyday lives. The VRE carriage was seldom considered during everyday activities, and it was not seen as a major issue compared with other illnesses. Those severely ill described that VRE would worry them more if otherwise healthy. However, informants described feelings of social exclusion and isolation due to carriership, though these feelings were more intense at the time of diagnosis. The informants shared their VRE status with close family, friends, and colleagues, often without being asked. Reactions involved questions on bathroom habits, comprehension, and seeking updated VRE information. However, a few informants chose not to disclose their status to others.

*‘I live the same way as usual … well, maybe I am a little more careful … I wash my hands and use hand sanitizer more often because they pointed out that it was quite important if you had this … it’s just things like that … otherwise, there is nothing special, really’* (informant 15).

#### Emotional strain

Informants expressed anger, fear, and anxiety about VREs, describing the carriage as an additional burden and stressor difficult to cope with, especially when they were already facing other health-related challenges.

*‘It’s an extra burden because I’ve had so much stress lately, so it becomes an additional stress factor … and when I’m trying to recover from this, it felt a bit like a slap in the face’* (informant 7).

Isolation policies during hospitalization, where they were confined to a private room without interaction, felt dehumanizing, akin to being managed as if they had the plague. This evoked a sense of stigmatization, feeling like ‘pariah’, or like carrying a permanent ‘mark’. Furthermore, informants felt that the healthcare system had given them an illness for life or sensed that it was their own fault. Additionally, the lack of compensation for VRE carriers contributed to feelings of frustration and unfairness.

*‘It is like you have a mark on you, so in certain moments, they [healthcare personnel] might understand, you’ve been hospitalized so many times and feel so bad; I was sick, so I had quite a lot of dark thoughts, and then this became another thing so that … um … I felt like I was walking around with a note that said “infected”’* (informant 21).

#### Performs hygiene measures in consideration for others

At home, the informants emphasized the importance of good bathroom hygiene, such as using personal towels, providing separate washcloths for visitors, and cleaning the bathroom thoroughly before having guests.

*‘I wash the toilet after I use it, and I had this kind of surface disinfectant that I had gotten hold of. So, I did my part. But they have to do their part and wash their hands thoroughly, and I had my own towels for them, for the visitors who come because they can’t get infected if they also wash their hands’* (informant 10).

One informant revealed that their partner used a separate bathroom, while another stated that they had stopped taking precautions after three negative tests. At work, they maintained strict hygiene practices, though some conceded that they had become less diligent over time. Public toilet use was another area of concern, frequently opting not to use them during hospital visits.

*‘yes, of course, then you were kind of thoughtful, and I know I had to work anyway, but I was careful about like this with gloves and hand hygiene and all that kind of stuff at work’* (informant 8).

## Discussion

VRE carriership had limited impact on daily life, though some experienced social exclusion. The informants reported unclear information and inconsistent management in healthcare and were reminded of their status during interactions with healthcare. Moreover, they had modified cleaning routines at home and were careful with their hand hygiene. Some were also afflicted with emotional distress.

VRE carriership had a minimal impact on daily life, with some informants being unaware of their status. In the interviews, ‘unaware’ referred to participants who reported that they had either not been informed about the result or did not recall having received such information. This aligns with previous findings, indicating unawareness of antibiotic-resistant bacteria carriage [6,[13], [14], [15], [16], [17]]. The finding may reflect challenges in how information about test results is communicated, for example, whether notification is provided through written correspondence, telephone contact, or during clinical visits. It also highlights the importance of clear and consistent communication processes. System-level strategies, such as standardized notification procedures and teach-back to confirm patient understanding, may help ensure that carriers are adequately informed.

Informants requested clearer information and chances to talk with healthcare personnel. Letters containing general information and/or phone calls were perceived as easily overlooked and insufficient. Similarly, Watson *et al.* [6] found that patients lack an understanding of their diagnosis due to insufficient information from healthcare providers, and healthcare personnel reported that they have insufficient knowledge about antibiotic-resistant bacteria. The failure to provide patients with adequate information could be due to limited knowledge of VREs among healthcare personnel [23] or inadequate health literacy [24], which is an obstacle to person-centred care [8,9]. A recent exploratory study reports that patients often face challenges in understanding written healthcare information [25], and Hamilton *et al.* [14] show that individuals desire tailored information and advice. Thus, concerns could be raised about the persistence of information gaps despite the implementation of person-centred care. Effective person-centred care requires meeting the patient at their health literacy level, as well as ensuring that the information is understood. Patients under contact isolation reported inadequate information about antibiotic-resistant bacteria and its impact. Addressing this gap requires communication training aimed at empowering patients and healthcare professionals.

Informants in the present study expressed concerns about persistent VRE carriage, transmission risks, and asymptomatic spread, which is consistent with existing research findings [12,13,17]. Worry about infecting others can cause emotional distress [12,16]. The lack of information provided to individuals regarding contagiousness may be because healthcare personnel also lack the right information. Hamilton *et al.* [14] emphasize the need for research on the accessibility and reliability of information for individuals with antibiotic-resistant bacteria. Notably, the lack of standard protocols for discontinuing VRE precautions in healthcare affects infectiousness assessments and ward screening practices [3]. While few informants experienced social exclusion, others reported occasional feelings of isolation. Hamilton *et al.* [14] also report such feelings and experiences and found that patients often feel misunderstood. This implies a need to further study how an antibiotic-resistant bacteria diagnosis affects individuals, regardless of whether they experience an active infection, to better meet patients’ need.

Informants described inconsistent VRE care practices, with private rooms that not only seclude the patient but also cause stigma and dehumanization. Door signage highlighting the presence of infection reinforced feelings of being labelled, which was also found in previous studies [6,13,14,17]. Stigmatization during hospitalization seems common and should be investigated further to reduce such feelings among patients. Informants felt distressed by being treated differently, a reaction also seen among individuals with other antibiotic-resistant bacteria [12,16,17]. When healthcare personnel avoid contact with patients, it can hinder the patient’s understanding of carriership and prevent the communication of other important information. Additionally, some healthcare personnel expressed a fear of contact with VRE carriers, which could explain their avoidance of the patient group. Tangeraas Hansen *et al.* [23] show that nurses are concerned about the risk of getting infected themselves. These findings emphasize the need for consistent, person-centred care that balances infection prevention and stigma reduction for individuals with VRE carriership.

Informants noted inconsistent protective equipment use by healthcare personnel, aligning with prior research on antibiotic-resistant bacteria and hygiene measures [6,[26], [27], [28], [29]]. Browning *et al.* [30] similarly documented variability among healthcare personnel in the management of VREs. However, hygiene guidelines clearly state when and how protective equipment should be used [4]. In the study by Tangeraas Hansen *et al.* [23], nurses acknowledged that they did not always adhere to hygiene protocols, attributing this to time constraints and a lack of available rooms. Differences in the way healthcare personnel use protective equipment may contribute to confusion about the seriousness of carriership, which has been demonstrated in an interview study by Baron *et al.* [12]. As highlighted in a literature review by Morgan *et al.* [7], patients with antibiotic-resistant bacteria experience negative outcomes due to contact precautions. Therefore, it is crucial to address and mitigate these harmful effects and determine what hygiene precautions are needed. These findings highlight the need for better training to manage the VRE and prevent its spread without negatively impacting carriers.

Emotional responses to VRE detection, such as anger, anxiety, and fear, described in the present study indicate a significant source of stress, also reported in prior studies on antibiotic-resistant bacteria [[13], [14], [15], [16], [17]]. In comparison with a similar study on ESBL carriage [17], VRE carriage appeared to have a lower psychological impact, e.g. depression. This difference may be attributed to the characteristics of the population, as VRE carriers tend to be older with more comorbidities. Additionally, VRE carriage is often identified through screening rather than clinical cultures, meaning individuals are less likely to experience illness directly related to the bacteria. Some perceived that the healthcare system had imposed a lifelong condition on them, while others internalized blame for acquiring the bacteria. This aligns with the study by Skyman *et al.* [13], indicating a need for healthcare providers to offer better support to individuals with antibiotic-resistant bacteria.

### Methodological considerations

Credibility, dependability, and transferability describe trustworthiness in qualitative research [22] and are considered in this study. The methodology clearly describes the setting and sampling, data collection, and data analysis in accordance with the research objectives, which increases the credibility of the study. A limitation of this study is the limited description of participant characteristics. Although additional demographic or contextual information could have strengthened the interpretation and transferability of the findings, these data were not collected as part of the original study and cannot be obtained retrospectively due to the constraints of the ethical approval. Participants had received their diagnosis between 1 and 10 years prior to the interview. This temporal variation may have influenced how experiences were recalled, introducing potential recall bias. In addition, changes in healthcare practices over time may mean that participants’ accounts reflect somewhat different clinical contexts, which should be considered when interpreting the credibility and transferability of the findings. The relatively short duration of the interviews can be considered a limitation. An interview guide ensured that similar questions were asked of all informants, which may have contributed to the relatively short duration of the interviews. Probing questions were adapted to informants’ responses and the interaction during the interviews and therefore varied somewhat between interviews and interview modes. The use of mixed interview modes (telephone, Skype, or in-person) may also have influenced interview length by affecting informants’ willingness to share experiences. However, informants chose the interview mode themselves and appeared comfortable, and shorter interviews were not associated with any specific mode. Rather, the shortest interviews were more likely related to informants’ limited awareness of their carriership, resulting in fewer reflections on the phenomenon under study. Despite the relatively short interviews, the study had a narrow aim and participants had direct experience of the phenomenon. The interviews provided concise yet relevant descriptions, contributing to sufficient information power [21]. The results are illustrated by quotes, which is seen as a strength and contributes to credibility. Interviews were also conducted within a limited time frame, which ensures dependability. To increase the credibility and dependability of the study, the researchers maintained a constant dialogue to reach agreement in the iterative analysis process. The method and approach are carefully described, and we have addressed transferability to other contexts.

This study adds new evidence about how individuals with VRE carriage experience their lives. The findings indicate that information about VRE carriage is unclear, contributing to uncertainty in healthcare contacts; however, carriership does not appear to have a major impact on daily life. Informants described changes in cleaning routines, increased hand hygiene emotional strain, and differences in how healthcare personnel used protective equipment, which also reminded them of their condition. To improve clarity for patients, healthcare personnel need greater knowledge of VREs and appropriate management practices, as well as a stronger focus on person-centred care in healthcare interactions.

## CRediT authorship contribution statement

**Karin Uggla:** Writing – review & editing, Writing – original draft, Visualization, Validation, Methodology, Investigation, Formal analysis, Data curation, Conceptualization. **Robin Razmi:** Writing – review & editing, Writing – original draft, Visualization, Validation, Methodology, Conceptualization. **Josef D. Järhult:** Writing – review & editing, Visualization, Validation, Methodology, Conceptualization. **Kati Knudsen:** Writing – review & editing, Writing – original draft, Visualization, Validation, Methodology, Conceptualization. **Maria Lindberg:** Writing – review & editing, Writing – original draft, Visualization, Validation, Project administration, Methodology, Formal analysis, Data curation, Conceptualization.

## Data availability statement

The participants in this study did not give written consent for their data to be shared publicly; so, due to legal restrictions, the data are not publicly available.

## Ethics statement

In accordance with national laws, the study was approved by the Swedish Ethical Review Authority (Reg. nos. 2022-03364-01 and 2022-05296-02) on 22^nd^ of December 2022. The study was conducted in accordance with the Declaration of Helsinki, and informed written consent for participation was obtained.

## Declaration of Generative AI and AI-assisted technologies in the writing process

AI or AI-assisted tools were not used in drafting any aspect of this manuscript.

## Funding sources

This research was funded by 10.13039/501100009776Region Gävleborg (grant number: CFUG-973237; CFUG-978656), University of Gävle (grant number not applicable), University of Uppsala (grant number not applicable), and the Family Olinder-Nielsen’s Foundation (grant number not applicable). The funding sources were not involved in the study design, in the collection, analysis and interpretation of the data, in the writing process, or in the decision to submit the manuscript for publication.

## Conflict of interest statement

The authors declare that they have no known competing financial interests or personal relationships that could have appeared to influence the work reported in this paper.
